# Convalescence

**Published:** 1875-02

**Authors:** 


### CONVALESCENCE.

The course of convalescence "never did run smooth." This is a general rule, as nearly unexceptionable as any maxim in medical science. The patient having passed the crisis of the disease, he begins to recover. But he seldom continues in an uninterrupted course of improvement. He is liable to "pull-backs," and that, too, in spite of the most vigilant care on his part and on the part of his attendants. Of course, if there is a known cause, it may be avoided in future. Perhaps a mistake has been made in the quality or quantity of the food taken; perhaps some undue exposure or a little fatigue may be justly charged as the cause of this interruption in the course of the convalescence. But we have often observed in recovery from the common diseases of this climate, perhaps more especially the continued fevers, pneumonia, pleurisy and intestinal complaints, that the patient has his good days and his sick days, or days in which he is not feeling quite as well. And these days *alternate*. To-day he is not as well as yesterday, and yet we cannot say that he is any worse. To-morrow he is all right again, and so he will continue, progressing favorably, on the whole, until his recovery is so nearly completed that the periodical days will be unnoticed.

But as no special cause is usually apparent, so no special treatment is required. Any anxiety on the part of the patient or his friends may be allayed by informing them of this characteristic in the natural progress of convalescence.

---

Geology of Cities.—From a hygienic point of view, the influence of geological formation is considerable. Cities built on rock are generally very salubrious; their impervious soil opposes the infiltration of water, which by natural slopes is carried far from the dwelling-places. The presence of clay, on the contrary, in the vicinity of foundations, is a cause of insalubrity, the waters from above gathering into pools and sheets in proximity to the upper stratum. The same happens in the case of sandy bottoms, when the layers of sand have little thickness. When the underground of a city is thus impregnated with moisture, it must be thoroughly drained. A complete drainage should not only leave dry the subjacent strata; it ought also, by the influx of fresh and pure air which it promotes, favor the oxidation of organic matter, the slow decomposition of which is often the cause of serious diseases. It has been ascertained in England that, since the underlaying of the town of Leicester, a diminution of forty per cent. took place in the mortality by phthisis. In Banbury and Salisbury it has been reduced by one-third and one-half respectively.

---

Five physicians of Lowell, Mass., have quit the Grand Army of the Republic because the surgeon of the post where they were members is a homœopath. The bigger fools they. If a physician can't survive the contact of one who declines to employ good-sized doses, preferring minute ones, he ought to abandon the profession. He may find five grains of "sub-murias" to be more effective than a hundredth part as much, but he is unworthy a place in the ranks of any "liberal" profession if he cannot allow his neighbor to employ the smaller dose without being heart-broken.

---

Only the weak or thoughtless man will deaden his intellect with alcohol, or tobacco, or opium.

